# ABCC1 Is Related to the Protection of the Distal Nephron against Hyperosmolality and High Sodium Environment: Possible Implications for Cancer Chemotherapy

**DOI:** 10.1371/journal.pone.0068049

**Published:** 2013-06-28

**Authors:** Leonardo M. Fonseca, Adriana B. Alvarez, Rachel C. Rodrigues, Diego H. F. Santos, Anibal G. Lopes, Marcia A. M. Capella

**Affiliations:** 1 Instituto de Bioquímica Médica, Universidade Federal do Rio de Janeiro, Rio de Janeiro, Rio de Janeiro, Brazil; 2 Instituto de Biofísica Carlos Chagas Filho, Universidade Federal do Rio de Janeiro, Rio de Janeiro, Rio de Janeiro, Brazil; Faculté de médecine de Nantes, France

## Abstract

**Aims:**

Glutathione (GSH) plays an important role in protecting cells against oxidative damage. ABCC1 protein transports GSH. Although this protein is largely studied in cancer, due to multidrug resistance phenotype, its role in the tubular cells of the kidney is unknown. The goal of this study was to find out whether ABCC1 has a role in protecting cells from the distal nephron against the stress caused by high medullar osmolality.

**Main Methods:**

MA104 cells were treated with high concentrations of sodium chloride, urea, or both to raise the osmolality of the culture medium. Cell viability was accessed by MTT and trypan blue assays. ABCC1 expression and extrusion of carboxi-fluorescein (CF), a fluorescent ABCC1 substrate, were measured by flow cytometry.

**Key Findings:**

Incubation of MA104 cells in a high sodium concentration medium resulted in changes in cell granularity and altered expression and activity of ABCC1. Urea did not alter ABCC1 expression or activity, but reversed the observed NaCl effects. High sodium concentrations also had a negative effect on cell viability and urea also protected cells against this effect.

**Significance:**

Our findings demonstrate that ABCC1 plays a significant role in the protection of kidney epithelial cells against the stress caused by high sodium environment present in renal medulla.

## Introduction

Multidrug resistance (MDR) is still the main cause of failure in cancer chemotherapy. Although MDR is multifactorial, it is primarily characterized by an ATP-dependent reduction in intracellular drug accumulation, due to the overexpression of three proteins belonging to the ABC transporters super family: P-glycoprotein (ABCB1), breast cancer protein (BCRP or ABCG2) or multidrug resistance-associated protein 1 (MRP1 or ABCC1). Although initially observed in tumor cells, these proteins are also present in normal cells. In kidneys, both ABCB1 and ABCG2 are expressed in the apical membrane of proximal tubules, suggesting a role in drug secretion [1], [2].

ABCC1, previously known as MRP1, is a transmembrane protein originally recognized as a transporter related to multidrug resistance phenotype in some cancer cells [3]–[6] but subsequent studies showed that this protein is ubiquitously expressed in virtually all organs in mammals, including humans [7], [8]. This transporter has a great importance in inflammatory processes and oxidative injury, given that it transports both reduced and oxidized glutathione, leukotriene C4 and prostaglandins [7], [9]. Its physiological role has been studied in several organs and systems and one of the most important discoveries is the fact that its presence is essential for the hypertensive response to angiotensin II [10].

However, concerning the physiological role of ABCC1 in kidney, it is not likely that this protein presents any sort of secretory role, due to the fact that it is restricted to the glomerulus and to the basolateral membrane of both the thick ascending limb of Henle’s loop and the medullar collecting duct [11]. That finding eventually postponed further studies concerning the importance of this transporter in kidneys, although some evidences point to a role of ABCC1 in urinary concentration mechanisms. For instance, thick ascending limb and distal tubule are especially sensitive to GSH depletion promoted by diethyl maleate (DEM), which forms complexes with GSH, reducing the intracellular levels of this tripeptide. Animals subjected to the treatment with DEM showed severe urinary concentration deficiencies [12], [13]. In addition, it was shown that etoposide induced polyuria in abcc1(−/−) mice, suggesting that this protein is somehow associated to water reabsorption [14].

Several anticancer drugs are known to induce nephrotoxicity. For example, the nephrotoxicity induced by cisplatin, a commonly used drug in anticancer therapy, limits its use in cancer treatment, and several research are performed to diminish this adverse effect [15]–[17]. Also, it was recently shown that nephrotoxicity associated to doxorubicin, a drug highly used in the chemotherapy of breast and other forms of cancers, is due to mitochondrial perturbations and cell death regulating genes [18].

Since nephrotoxicity is associated with several anticancer drugs [19], [20], and since the role of ABCC1 in kidney remains unsolved, the present study aimed to recognize a possible role of this protein in renal cells. We observed evidences that ABCC1 is related to the protection of the distal nephron against hyperosmolality due to a high sodium environment and that GSH is important to maintain the viability of distal nephron cells.

## Materials and Methods

All animal procedures were previously reviewed and approved by the Animal Subject Committee of the Centro de Ciências da Saúde - UFRJ with protocol number IBCCF 082/2009.

### Cell Culture

Monkey embryo cell line MA104, pig kidney epithelial cells LLC-PK1 and dog kidney epithelial cell line MDCK were all obtained from Rio de Janeiro Cell Bank. The cells were cultivated at 37°C in Dulbecco’s Modified Eagle Medium (Gibco, USA) with penicillin and streptomycin (Gibco, USA) and supplemented with 10% fetal bovine serum (Gibco, USA) and L-glutamine (Gibco, USA).

### Treatment with Osmolytes

To raise osmolality of the culture medium, 100 mM NaCl, 200 mM urea, 200 mM mannitol, or 50 mM NaCl +100 mM urea (final concentrations) were added to the medium, raising its osmolality to approximately 450 mOsm/Kg H_2_O. The solutions were added either gradually, over a period of 72 hours, or in a single addition, as described in the figures’ captions.

### MTT Assay

MTT colorimetric assay [21] was used to assess the viability of the MA104 cell line incubated in hyperosmotic medium. Approximately 2×10^4^ cells per well were seeded onto 96 wells plates. The next day NaCl, urea, mannitol or NaCl+urea were added to the medium, as described above. In some experiments Buthionine–Sulfoximine, an inhibitor of GSH synthesis (BSO-Fluka, USA), and MK571, an ABCC1 inhibitor, were also added. After incubation periods of 24, 48 or 72 h, 20 µL of 10 mg/mL Thiazoyl Blue Tetrazolium Bromide (Sigma, USA) were added to the culture and the cells were further incubated for 3 h at 37°C. At the end of the incubation time the supernatant was discarded and 100 µL of Dimethyl Sulfoxide were added per well to dissolve the newly formed formazan salt. The absorbance was measured at 490 nm using an ELISA plate reader (Benchmark, BioRad). Mean values were calculated from 3 independent experiments.

### Trypan Blue Staining

Another method used to access cell viability was cell counting with Trypan blue staining. In this case, 1×10^5^ cells/well were seeded onto 24 well plates and after incubation with the osmolytes as described above, the cells were counted on a hemocytometer.

### Glutathione

A commercial kit using monochlorobimane as substrate (Millipore, USA) was used to measure glutathione (GSH) levels. For this assay, 1×10^6^ cells were seeded onto culture bottles, treated as above, and harvested with trypsin. GSH assay was performed according to the manufacturer’s instructions. Fluorescence levels were read using a plate reader with a 380/460 nm filter set.

### ABCC1 Activity

Cells were seeded onto 24 wells plates and treated with osmolytes as described above. Carboxy-fluorescein diacetate (CFDA – Molecular Probes, USA) is a non fluorescent molecule that is converted into a fluorescent one, carboxy-fluorescein (CF) by intracellular esterases. This molecule is a substrate for ABCC1. Therefore, to determine ABCC1 activity, the cells were incubated for 30 minutes with 2 µM CFDA (to permit dye uptake, esterase action, and accumulation of CF), rinsed with phosphate buffered saline (PBS), to wash out the dye and incubated for another 30 minutes in CFDA-free DMEM for CF extrusion. The cells were then harvested with trypsin, washed and ressuspended in saline. Intracellular fluorescence was measured in a Beckton-Dickinson flow cytometer (FACSCalibur). Data analysis was performed by the software Summit (Dako Inc, USA).

### Labeling with Anti-ABCC1 Antibody

Cells were seeded onto 24 wells plates and the labeling assay was performed as described in a previous work from our group [22]. The polyclonal rabbit antibody A23 (Alexis Biochemicals, USA) and Alexa Fluor 488 (Invitrogen, USA) were used respectively as primary and fluorescent secondary antibodies.

### Western Blotting Analysis for Tamms-Horsfall Protein (THP or Uromodulin) and Aquaporin 2 (AQP2)

Expression of THP and AQP2 was assessed by immunoblotting using specific antibodies. Cells were plated onto 6-well plates at a 5×10^5^ cells/well and incubated at 37°C. After 48 hours of incubation, the culture media was aspirated; the cells were washed three times with PBS (pH 7.4) at room temperature, scraped, and centrifuged at 14000 ***g*** for 60 seconds. The cells were then suspended in sample buffer (Trisma base 0,76% m/v; glycerol 12,56% m/v and sodium dodecyl sulphate 2,3% m/v; pH 6,8). After homogenization, a small sample was used for protein measurement. The buffer was then complemented with 0.6% dithio-L-threitol (DTT), 0.5% β-mercaptoethanol, and bromophenol blue (1 mg/mL). The proteins were then subjected to SDS-polyacrylamide gel electrophoresis (PAGE) and transferred to a PVDF membrane (Bio-Rad, Hercules, CA, USA). The membranes were blocked with Western Breeze blocking solution (Invitrogen, USA) and incubated with specific antibodies against THP or AQP2. The phosphatase alkaline Western Breeze Kit (Invitrogen, USA), was used to visualize the bands in the membranes. The subclone C7 from MDCK cell line was used as a positive control for AQP2, since it is composed of principal cells from the collecting duct [23]. Other controls were extracts from rat kidney medulla and cortex. For this, Whistar rats had their kidneys removed, dissected and stored at −80°C for processing. The organs were then homogenized in a solution composed of PMSF (1 mM), sucrose (250 mM), EDTA (1 mM) and imidazole (20 mM) until a homogenate was obtained.

### Statistical Analysis

Each experiment was repeated from three to five times. Data were expressed as means ± standard deviation of the mean and were analyzed using Students t-test or one-way ANOVA with Dunnet or Bonferroni post tests for comparison of the differences. Values of P less than 0.05 were considered statistically significant.

## Results

### Characterization of MA104 Cells

**Figure 1:**
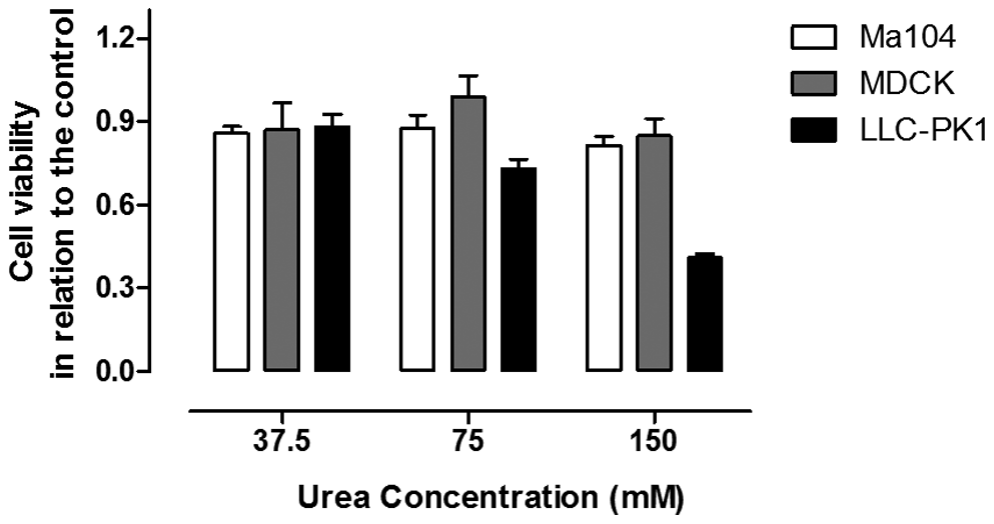
Sensitivity of three renal cell lines from different origins to urea. The cells were seeded onto 24-wells plates and urea was added 24 h later in several concentrations. After 48 h of incubation, the cell number and viability were measured by Trypan Blue staining (n = 4). a – different from 37.5 mM (p<0.01); b – different from 75 mM (p<0.001).

Since there is no human cell line from distal nephron commercially available, we tried to test whether the renal monkey cell line MA104 was originated from distal nephron. First, we compared the sensitivity of three mammal renal cell lines to urea: MDCK, a canine kidney cell line with characteristics of distal nephron [24]–[26]; LLCPK1, a porcine renal cell line with characteristics of proximal tubules [27] and MA104, not yet characterized. Cells were incubated for 48 h with various urea concentrations and the cell number and the cellular viability were measured by Trypan Blue staining. As can be seen in Fig. 1, MA104 cells are as resistant to urea as MDCK cells, while LLC-PK1 cells are more sensitive.

**Figure 2:**
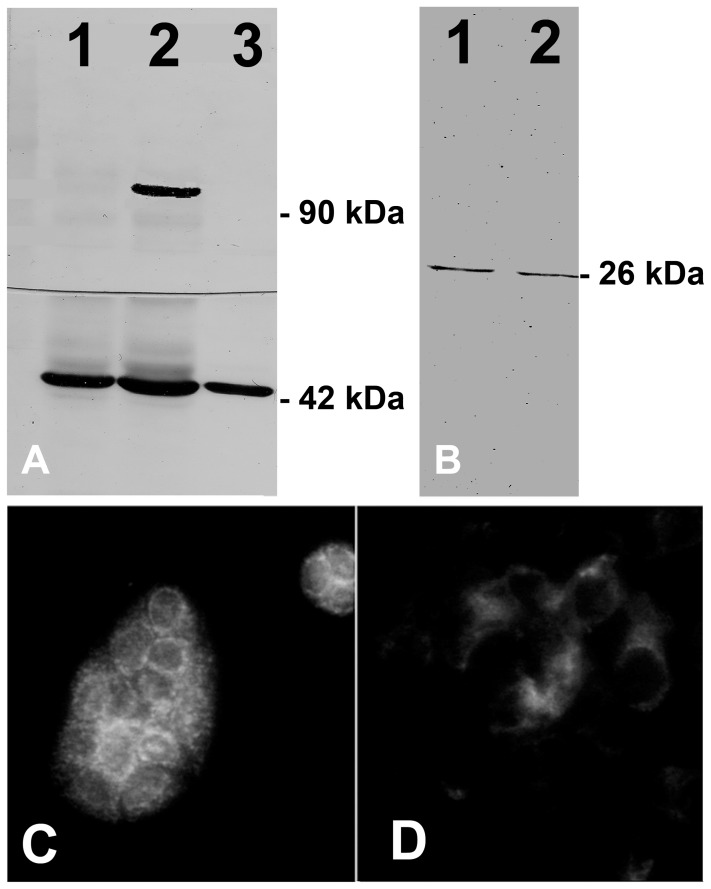
Characterization of the MA104 cell line. A. Expression of THP protein in rat kidney cortical (lane 1) and meldullary (lane 2) extracts and MA104 (lane 3) in the upper membrane and β-actin in the lower membrane; B. Expression of Aquaporin 2 in MDCK-C7 (lane 1) and MA104 (lane 2); C and D. PNA labeling (100 µM) of MDCK-C11 (C) and MA104 (D).

MDCK is a dog kidney cell line and there are considerable differences between dog and humans. Moreover, several antibodies raised to humans do not label dog proteins. On the other hand, MA104 is a monkey kidney cell line, and it is well accepted that non human primates share numerous characteristics with humans. We have previously shown that antibodies against human ABCB1 and ABCC1 also labeled this cell line [28], [29]. Given that ABCC1 is expressed only at the basolateral membranes of distal straight tubule and the collecting duct epithelia [11], and taking into account the resistance of MA104 cells to urea, it seems likely that these cells came from distal nephron. Therefore, we evaluated some characteristics of distal nephron segments in this cell line. Our results showed that MA104 cells did not show significant labeling with PNA, a marker for intercalated cells and did not express uromodulin, a protein that is expressed only in the distal straight tubule, but express AQP2, a protein characteristic of principal cells of the collecting duct (Fig. 2). Taken together, these results suggest that MA104 cells are originated from collecting duct, making them a good model to study the possible role of ABCC1 in the resistance of kidney cells to hyperosmolality.

### Sensitivity of MA104 Cells to Several Osmolytes

**Figure 3:**
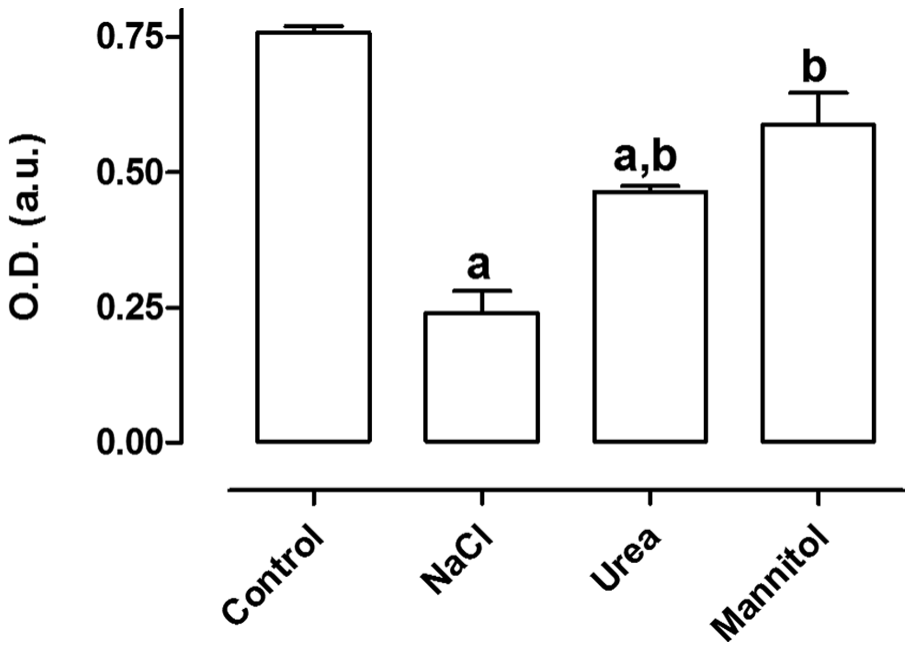
Effect of different osmolytes on the viability of MA104 cells. After an initial incubation of 24 hours at 37°C, osmolytes were added to the culture medium and the cells were incubated for additional 48 hours. Results are expressed as average ± SE (n = 3). a – different from control (p<0.05); b – different from NaCl (p<0.01).

The next step was to determine the effect of different osmolytes in the survival of MA104 cells. Medium osmolality was raised with sodium chloride, urea or mannitol in a single addition (an osmotic shock) and the viability was measured by MTT assay. The concentration of NaCl used was previously determined as the minimum NaCl concentration to achieve maximum effect on cell survival (data not shown). Fig. 3 shows that addition of sodium chloride greatly reduced cell survival, whereas urea and mannitol had only a small effect. This result suggests that the reduced viability of cells treated with NaCl is not due to hyperosmolality itself, since mannitol did not produce the same effect.

**Figure 4:**
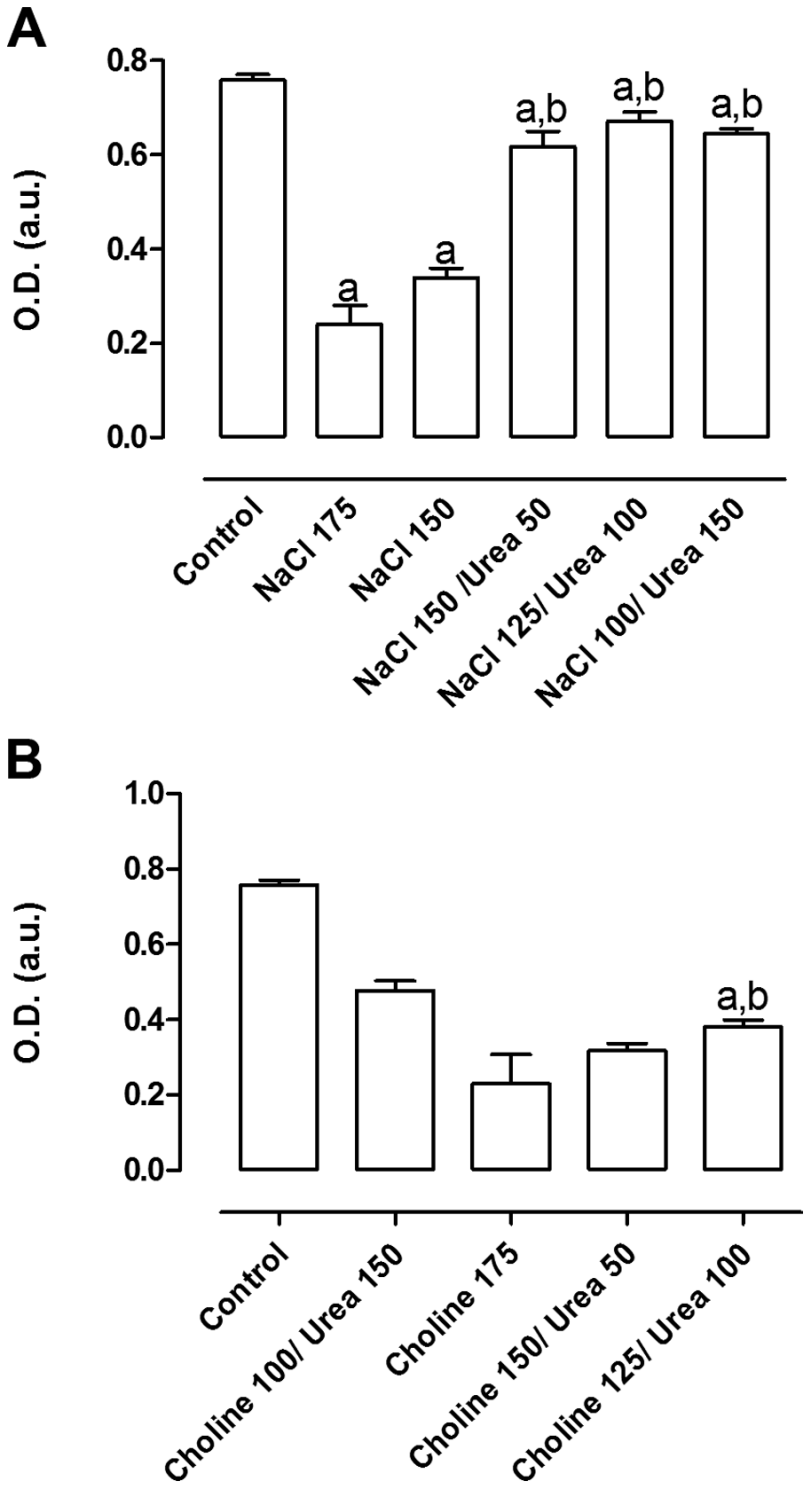
Protection of urea against the reduction in viability of MA104 cells treated with high sodium concentration. After an initial incubation of 24 hours at 37°C, osmolytes were added to the culture medium and cells were incubated for additional 48 hours; A. Cells treated with NaCl or NaCl+urea. Results are expressed as average ± SE (n = 3). a – different from control (p<0.05); b – different from NaCl 175 mM (p<0.001) and B. Cells treated choline chloride (shown as choline) or choline chloride+urea (n = 3). a – different from control (p<0.001); b – different from choline 175 mM (p<0.001).

Since NaCl may affect several functions, such as sodium channels, chloride channels, sodium-potassium ATPase etc, and since there is no data in the scientific literature suggesting whether Na^+^ or Cl^−^ is the main cause of the decrease in cell viability, we decided to study this point. For comparison, we used choline chloride, which maintains the same ionic force of the medium whithout the presence of Na^+^. In Fig. 4 is shown that NaCl and choline chloride equally reduced cellular viability, suggesting that the chloride ion bears the responsibility for this effect.

### Effect of Urea in the Sensitivity of MA104 Cells to NaCl and Choline Chloride

Some authors observed that urea protected cells against injury induced by sodium chloride [30]. As medullary hyperosmolality in the kidney is mainly due to urea and NaCl, our next step was to investigate a possible protective effect of urea on cell viability under high NaCl concentration. Cells were then submitted to an osmotic shock with NaCl in the presence or absence of urea. In order to maintain the same osmolality of the medium, different amounts of NaCl and urea were used (the final osmolality of the medium with 50 mM urea +150 mM NaCl was similar to that of medium added with 175 mM NaCl alone, roughly 561 mOsm/Kg H_2_O as measured by an osmometer). For comparison, we also used choline chloride. It can be seen in Fig. 4 that urea protected cells submitted to high NaCl, but the effect of urea in cells treated with choline chloride was much less than that observed for NaCl. This suggests that both ions (Na^+^ and Cl^−^) participate in cell death, but the effect of urea is more pronounced under a NaCl environment, which is in accordance to the renal medullar environment. Since choline chloride is not an osmolyte in renal medulla, we did not use this substance in the following experiments.

### Intracellular GSH Levels in MA104 Cells Treated with Osmolytes

**Figure 5:**
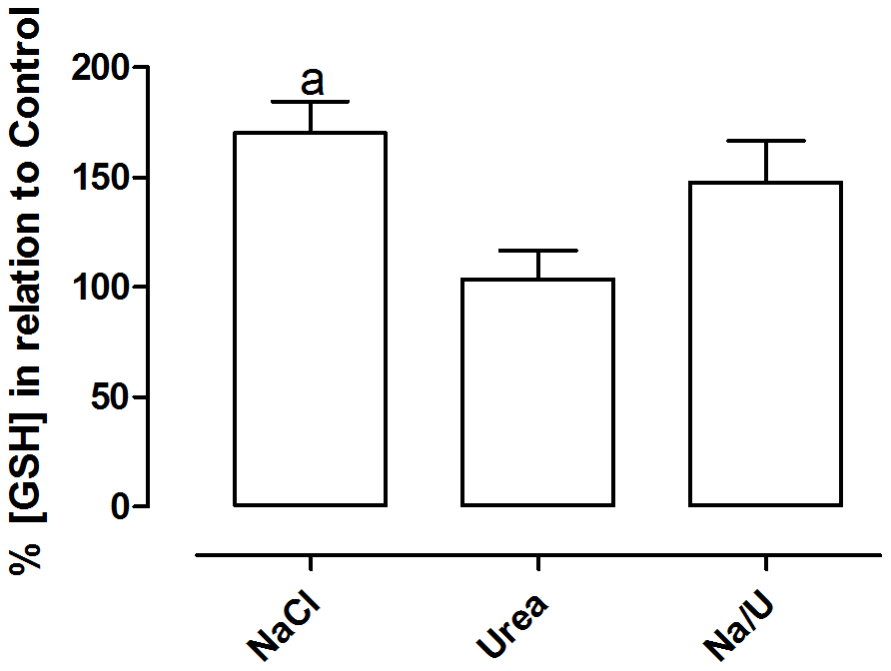
Effect of hyperosmolality on reduced glutathione levels. Cells were incubated as indicated in Figure 4 and the intracellular levels of GSH were measured as described in material and methods. Results are expressed as average ± SE (n = 8); p<0.05 (a - different from both control and urea).

**Figure 6:**
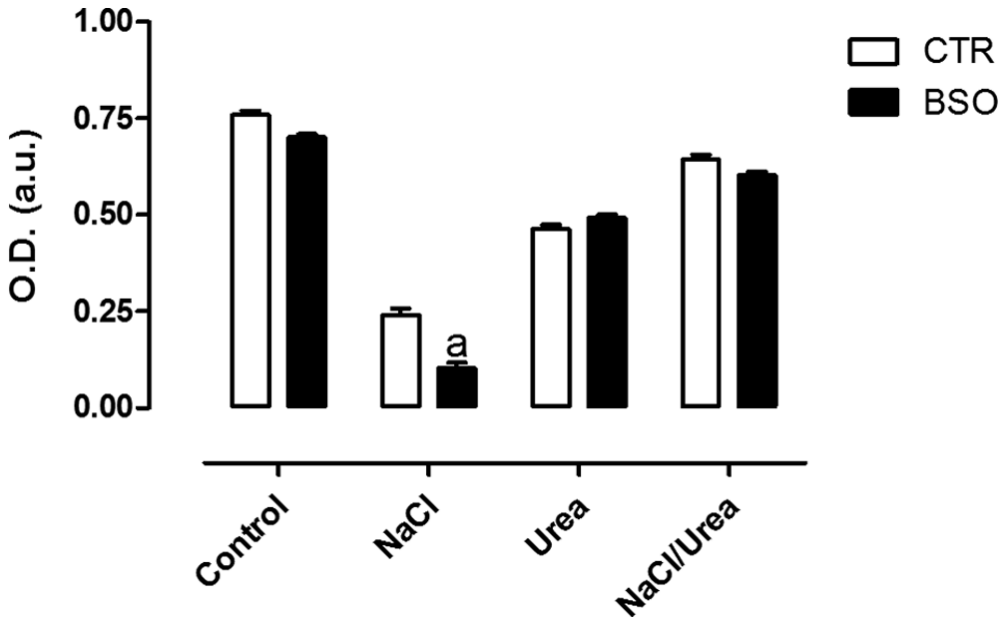
Effect of BSO on the reduction in cellular viability induced by high sodium concentration. After an initial incubation of 24 hours at 37°C, BSO (1 mM) was added to the culture medium and after 24 hour of incubation osmolytes were added to the culture and the cells were incubated for further 48 hours. Results are expressed as average ± SE (n = 3); p<0.05. (a - different from NaCl).

There are several evidences that NaCl lead to oxidative damage [31], [32]. Since GSH is one of the most important intracellular molecules related to the protection against oxidative stress [33], the levels of intracellular GSH were evaluated in MA104 cells after treatment with the osmolytes. For this assay, the osmolytes were added in a gradual manner, over the course of 72 hours and the content of GSH was measured. In Fig. 5 is shown that treatment of cells with NaCl caused a significant increase (approximately 50%) in the intracellular levels of reduced GSH in MA104 cells. Urea alone did not alter the intracellular content of GSH, but reversed the GSH increase observed in cells treated with NaCl. To test whether this elevation in GSH levels could be related to cell survival, we used BSO, an inhibitor of glutathione synthase, which would impair this elevation in GSH levels. The cells were pre-incubated with BSO before treatments with NaCl or urea. As expected, inhibition of GSH synthesis by BSO reduced by 50% the survival of cells treated with NaCl, but did not alter that of cells treated with urea or urea+NaCl (Fig. 6), suggesting that cell death in the presence of urea+NaCl occurs by a different way than that with NaCl alone and is not dependent on GSH.

### Expression of ABCC1 in MA104 Cells Treated with Osmolytes

**Figure 7:**
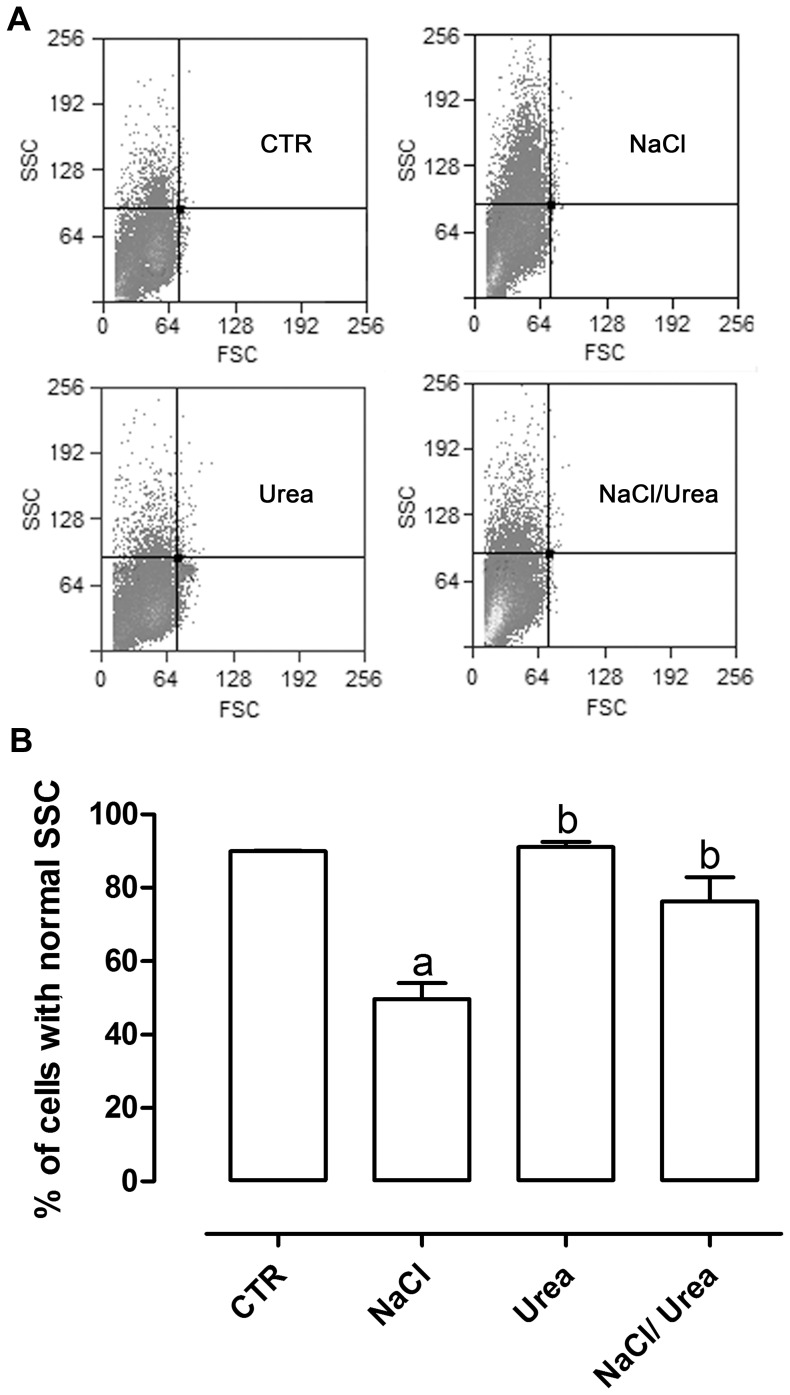
Effect of hyperosmolality on cell volume and granularity. Cells were incubated during 96 hours at 37°C with the osmolytes. A - dot plots showing the difference between forward scattering (FSC) and side scattering (SSC), representing respectively cell volume and granularity. B – Percentage of cells exhibiting normal granularity for each treatment group. Results are expressed as average ± SE (n = 4); (a - different from control; p<0.001 and b- different from NaCl; p<0.01).

**Figure 8:**
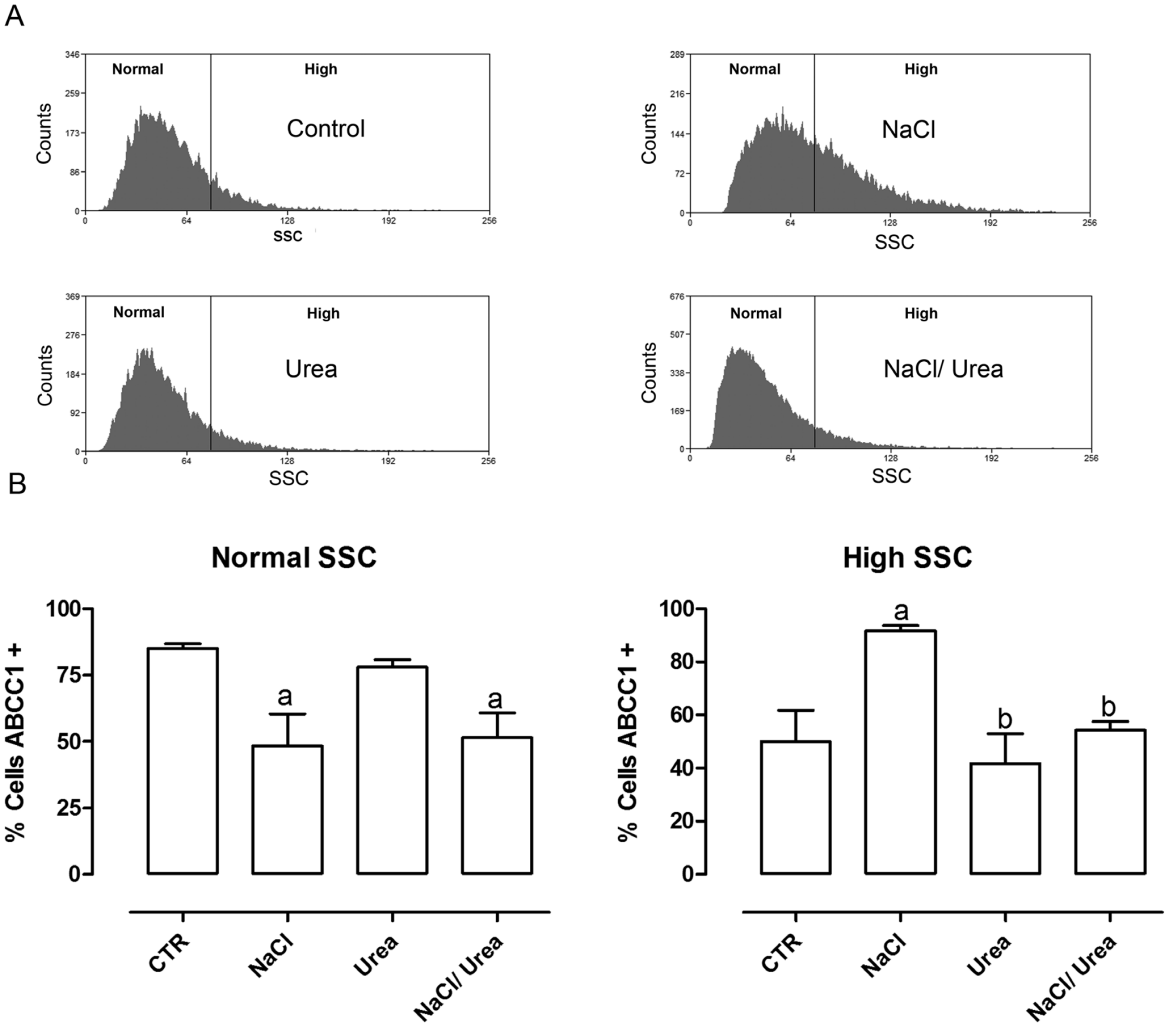
Effect of hyperosmolality on the percentage of ABCC1-positive cells in the subpopulations. Cells were incubated with the osmolytes during 96 hours at 37°C. The cells were then labeled with anti-ABCC1 antibody and the expression of this protein was analyzed by flow cytometry. Each treatment group was divided into two subgroups according to SSC values. A – Histograms showing the two subpopulations (normal and high SSC) for each condition and B – Percentage of ABCC1-positive cells from both normal and high SSC subpopulations. Results are expressed as average ± SE (n = 5). a – different from control (p<0.01) and b- different from NaCl; p<0.01).

Since the maintenance of intracellular GSH levels is closely associated with ABCC1 transport [34], [35] and given that MA104 and collecting duct cells express ABCC1 [14], [22], in the next experiments we tried to study a possible role of ABCC1 in the response of MA104 cells to NaCl and urea. However, when performing flow cytometry, we observed that NaCl lead to alterations in cell granularity (viewed by alterations in side scattering-SSC), with no changes in cell volume (viewed by alterations in forward scattering-FSC) as can be seen in Fig. 7A. Therefore, we first studied such alterations. To do this, the cells were separated in two regions: region R1, containing the cells with normal SSC and region R2 containing the cells with high SSC (Fig. 8A).

In Fig. 7B is shown that 90% of the control cells have normal SSC and that treatment for 72 h with urea alone did not alter cell granularity. However, NaCl greatly increased this parameter, with nearly half the population having greater SSC. Moreover, urea reversed this alteration, since 76% of the cells submitted to NaCl+urea was in the region with normal SSC and this was not statistically different from control cells. Although this increase in cell granularity could be due to a reduced cell volume, due to drop of cell water content, we could not observe this. The FSC of cells submitted to all treatments were similar to that of control cells, suggesting no alteration in cell volume after prolonged treatment with NaCl, Urea or NaCl+Urea (Fig. 7A).

In view of the observed effects of NaCl treatment in SSC, for the study of ABCC1 expression and activity we separated the cells in two regions, one related to cells with normal SSC and the other related to cells with high SSC. The results depicted in Fig. 8 showed that, for cells with normal SSC, approximately 85% of the control cells express ABCC1. This percentage is reduced to approximately 50% in cells treated with NaCl, and urea did not reverse this. For cells with high SSC, the percentage of cells expressing ABCC1 is roughly 50% in control cells. NaCl increased this to approximately 92%, and urea altered this, returning the values close to the control cells (54%).

**Figure 9:**
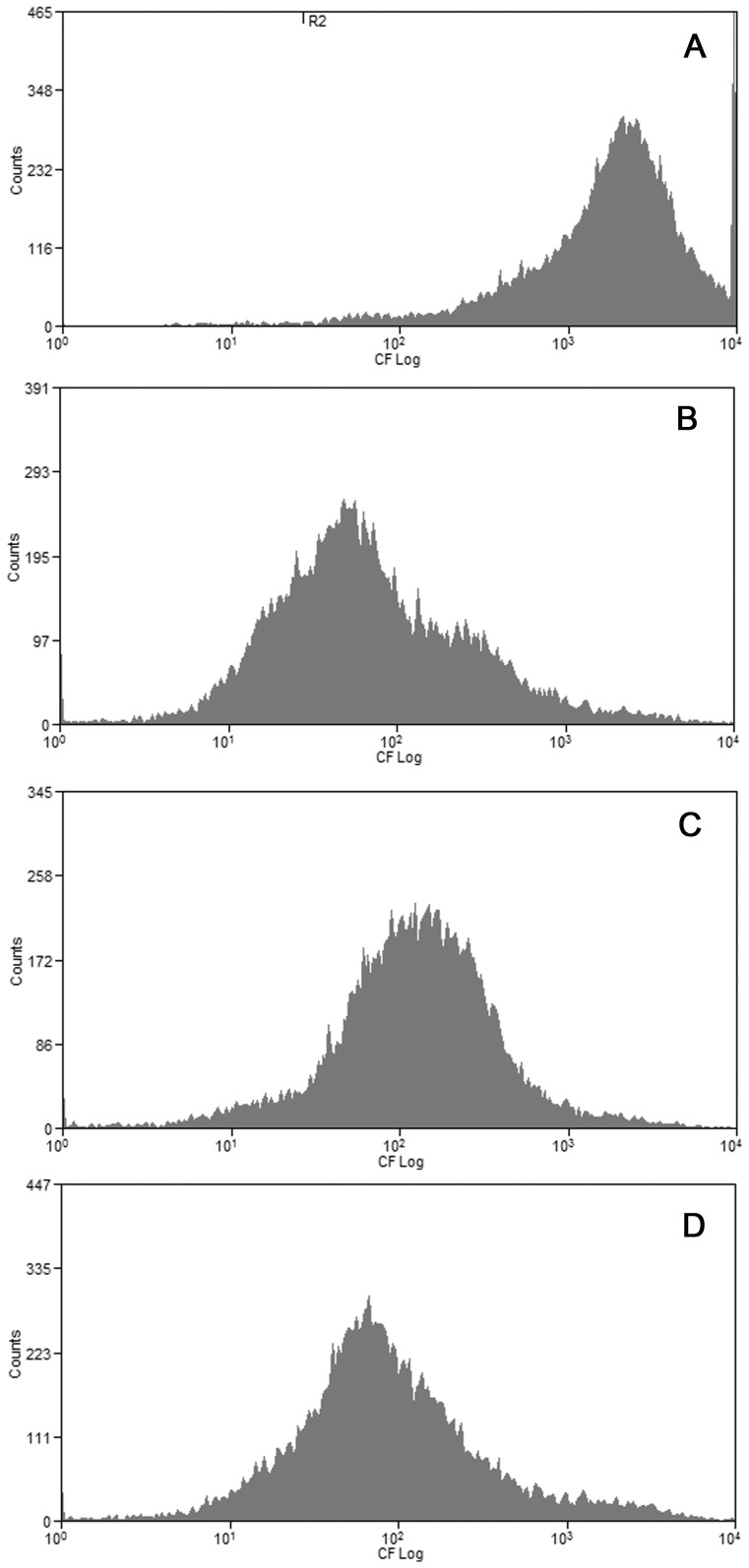
Effect of hyperosmolality on the accumulation of CF. Total population of cells was incubated during 96 hours at 37°C with the osmolytes and the cellular fluorescence, showing the accumulation of CF after 30 min of incubation was measured as described in Material and Methods. Panel A shows the accumulation of CF in control cells, as evidenced by the high fluorescence. Panels B, C and D show the accumulation of CF in cells treated respectively with NaCl, urea or NaCl+urea. Histograms representative of 3 different experiments.

**Figure 10:**
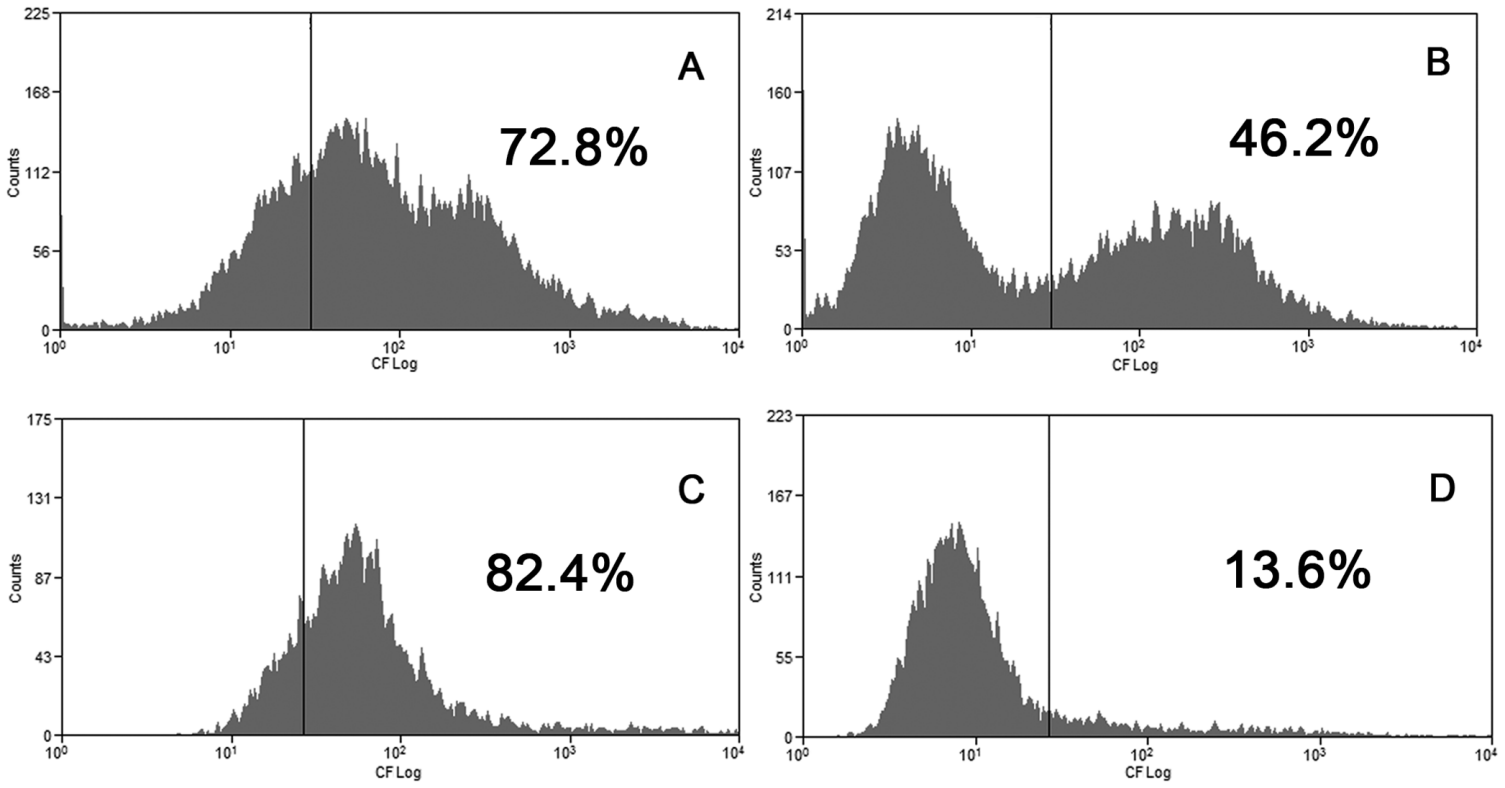
Effect of treatment with NaCl on ABCC1 activity. Cells were incubated with NaCl during 96 hours at 37°C and the activity of ABCC1 was measured as described in Material and Methods. Upon analysis, the cells were divided into two subgroups according to the side scatter values. Left panels: CF accumulation by cells with normal (A) and high (C) SSC; Right panels: CF efflux from cells with normal (B) and high (D) SSC. Histograms representative of 3 different experiments. The values in each panel refer to the percentage of cells with fluorescence, which means cells that accumulate CF (in panels A and C) and cells that were not able to efflux the dye after 30 min in dye-free medium (in panels B and D).

**Figure 11:**
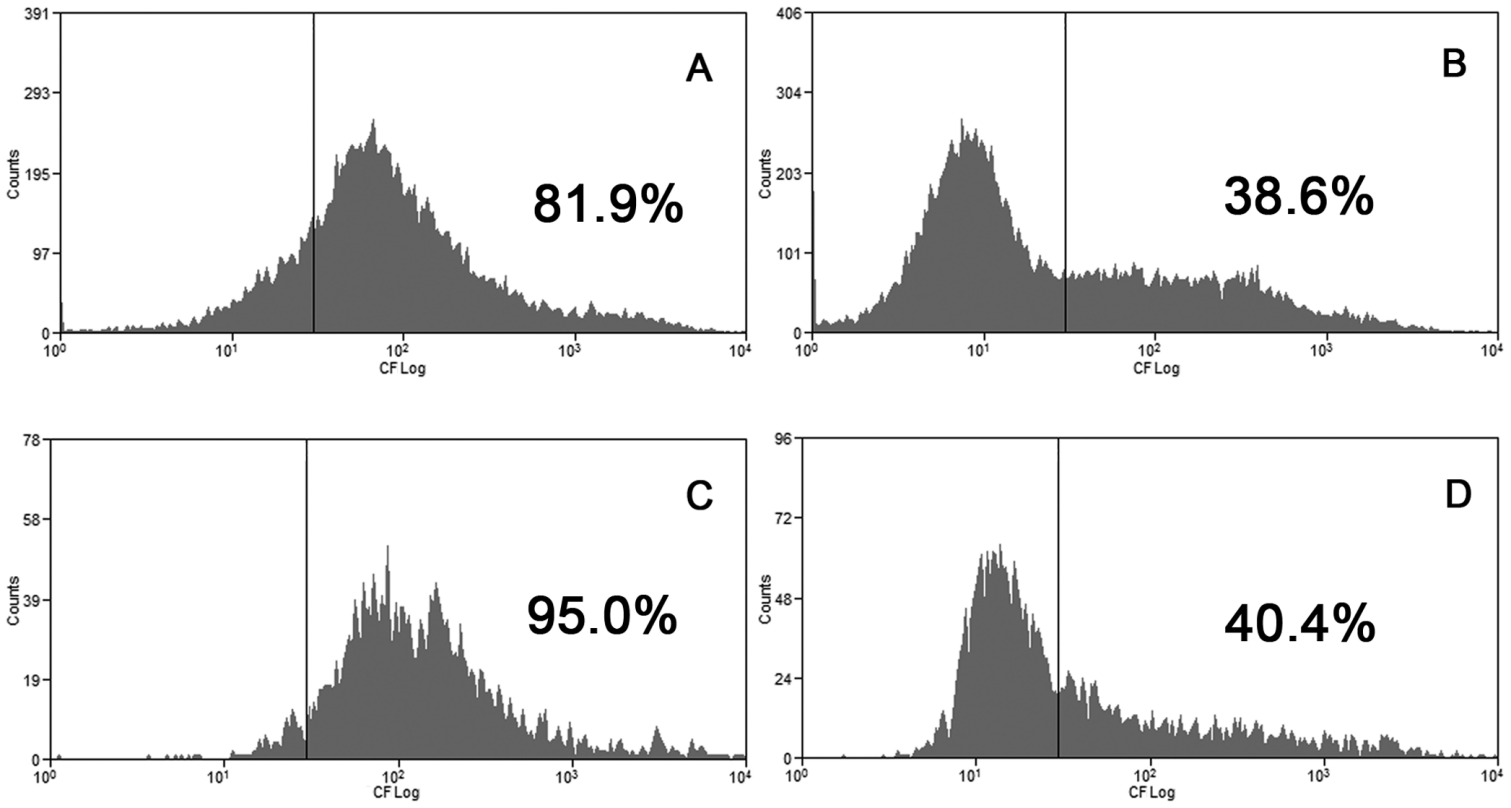
Effect of treatment with NaCl+urea on ABCC1 activity. Cells were incubated with NaCl+urea during 96 hours at 37°C and the activity of ABCC1 was measured as described in Material and Methods. Upon analysis, the cells were divided into two subgroups according to the side scatter values. Left panels: CF accumulation by cells with normal (A) and high (C) SSC; Right panels: CF efflux from cells with normal (B) and high (D) SSC. Histograms representative of 3 different experiments.

The next step was to study the ABCC1 activity by flow cytometry. When doing this, we observed that control cells accumulate much more CF (a common used substrate for ABCC1) than cells treated with osmolytes, probably because the hyperosmolality of the media with NaCl, urea and NaCl+urea hindered the cellular uptake of the dyes (Fig. 9). Although this impaired the comparison of osmolyte-treated cells with control cells, we could still compare cells with normal or high SSC inside the same treatment. Therefore, in Fig. 10 is shown the accumulation and the efflux of NaCl treated cells with normal (Fig. 10A and 10B) and high (Fig. 10C and 10D) SSC. It can be seen that the cellular uptake of CFDA was similar regardless of normal or high SSC (Fig. 10A and 10C). However, the efflux of the fluorescent dye by cells with high SSC was almost complete (Fig. 10D), whereas in cells with normal SSC approximately 50% of the cells did not efflux the dye (Fig. 10B). On the other hand, cells treated with NaCl+urea were equally able to efflux the dye, regardless normal or high SSC (Fig. 11b and 11d).

## Discussion

MA104 is a cell line derived from kidney of African Monkey embryos [36]. It expresses several characteristics of polarized epithelial cells, such as the formation of “domes”, the establishment of a transmonolayer electrical resistance and polarized maturation of enveloped virus [37]. We have previously shown that this cell line expresses at least two proteins related to multidrug resistance, ABCB1 and ABCC1 [28], [29], [38]. In the present study, we further characterized these cells, and it seems very likely that MA104 originated from collecting duct, probably principal cells. Such characterization is important to establish this cell line as a model for study of ABCC1 in the kidney.

Once characterized, we showed the rise in osmolality due to NaCl or choline chloride (but not urea) caused a decrease in cell growth. Moreover, urea protected cells from growth inhibition or cell death induced by NaCl but not choline chloride (Fig. 3–4). These results are in accordance with other authors who showed that urea could reverse the deleterious effect attributed to NaCl [30]. It has also been shown that, although urea can induce oxidative injury, it is also capable of activating protective mechanisms against such oxidative damages, and that NaCl also activated protection mechanisms, but only when it was gradually added to the medium [39], [40]. However, the mechanisms by which such effects occur are still not understood. The exact process through which NaCl causes reduction in cell viability is yet unknown. Some authors seem to agree that high NaCl concentrations cause cell cycle arrest. Both Michea and Kultz claim that mIMCD suffer cell cycle arrest in G2 after incubation with high sodium chloride concentrations, leading to reduced proliferation [41], [42]. Dimitrieva adds to that claim, showing that p53 levels rise rapidly in mIMCD3 cells after incubation with sodium chloride, reducing cell proliferation at first moment [43]. A later study again suggests that NaCl induces a reduction in proliferation, now due to an increase in egr-1 expression, which would serve as a stimulus to differentiation [44]. Kultz [45] further claims that NaCl in high concentrations leads to double strand breaks in DNA, which could be caused by free radicals. Furthermore, Zhou and Zhang agree that high NaCl causes an increase in ROS and consequently oxidative damage [46], [47]. As it stands, evidence seems to point that reduction of viability tends to point toward oxidative stress as a starting point.

The protection offered by urea is as controversial as is NaCl induced damage. One study suggests that urea itself leads to rise in ROS, but also is capable of inducing the expression of heme oxygenase-1 (HO-1) in kidney cells, which could comprise part of the adaptive or protective system to hyperosmolarity [40]. Zhang et al, shows that addition of urea to NaCl treated cells leads to inhibition of caspase-3 activity [30]. Also, Santos showed that inner medulla kidney cells treated with both NaCl and urea showed an increase in HSP70 that correlates to the increase in osmolarity. The authors suggest that such finding, associated with the proliferation reduction seen in the treated cells could be part of the protection mechanism that enables cells to survive in the kidney medulla [48]. Taken together, the data suggests that while NaCl (and in some models, urea) leads to increase in ROS and consequent DNA damage, a concomitant treatment with urea leads to the activation of response mechanisms such as chaperone expression that leads to DNA protection. However, our results showed that the protection of urea is against damage induced mainly by Na^+^ ion and not necessarily NaCl. This suggests that new investigations are needed in order to understand both the lethal effect of NaCl and urea and the interaction between those two components in renal medulla.

Given that GSH is the main non-enzymatic antioxidant molecule, we studied the intracellular GSH levels of cells treated with NaCl and/or urea. It was observed that NaCl, but not urea, increased the intracellular GSH levels and that urea reversed the increase in GSH levels in NaCl-treated cells (Fig. 5). The increase in GSH content in NaCl treated cells could be due to inhibition of ABCC1 activity or expression, but could also be due to an increase in oxidative stress. Moreover, BSO reduced by 50% the survival of cells treated with NaCl, but did not alter the survival of cells treated with urea or urea+NaCl (Fig. 6). These results suggest that GSH is important for cell survival in the presence of high NaCl concentrations, but not in the presence of urea, and that the mechanism of urea protection may be related to the regulation of oxidative stress, which in turn regulates intracellular GSH levels.

Interestingly, this result is opposite to that observed by Zhang et al. [30]. Those authors showed that urea reduced GSH content in mIMCD3, a murine renal medullary collecting duct cell line. This difference could be due to the cell species used or to the experimental conditions. In the present study, the cells were adapted gradually to hyperosmotic conditions and the intracellular GSH level was measured after 72 h, while in the study of Zhang et al. the cells were treated only for 1 h.

The results discussed above suggest that the mechanisms of urea protection are probably not related to GSH itself. Since the transport activity of ABCC1 depends directly on intracellular GSH levels [49], it is possible that the modulation of ABCC1 activity is an important factor regulating GSH levels, especially in situations where high levels of GSH are required for cell survival. Therefore, we tested the expression and activity of ABCC1 in cells treated with NaCl, urea or NaCl+Urea. An unexpected result was that NaCl altered the cell granularity (SSC) without alteration in cell volume (FSC) (Fig. 7). In normal SSC cells, roughly 85% of the cells express ABCC1, whereas in high SSC cells, only 50% cells express ABCC1. Interestingly, the two populations behaved differently towards the treatment with NaCl, urea and NaCl+urea. In normal SSC cells, the treatment with NaCl significantly reduced the percentage of the ABCC1 positive cells, while in high SSC cells, the same treatment significantly increased the percentage of ABCC1 positive cells. Therefore, NaCl seem to have different effects in this cell line and we believe this is an interrogation for future studies. It is possible, for example, that MA104 is not a homogeneous culture, as occurs with MDCK and other renal cell lines from distal nephron, where principal and intercalated cells coexist [23]. Although our results showed that the majority (90%) of the cells have characteristics of principal cells, it is possible that the 10% of cells which have high SSC in control may be a subpopulation of intercalated cells, and it is known that intercalated cells respond differently from principal cells to NaCl [50]. The ions Na^+^ and Cl^−^ may be transported by several different proteins, such as the Na^+^/K^+^/2Cl^−^-cotransporter NKCC2 in the straight distal tubule, the Na^+^/Cl^−^-cotransporter NCC in the distal convoluted tubule, or the epithelial sodium channel (ENaC) expressed in principal cells. Chloride may be reabsorbed either by a paracellular pathway that may be controlled by claudins or the transcellular route through non-type A intercalated cells, which express on their luminal membrane the anion exchanger pendrin (SLC26A4). The type A intercalated cells, a third type of cells existing along the collecting duct, are critical for renal acid excretion and is classically not linked to blood pressure regulation. Although Na^+^ is long known as the responsible for salt-sensitive hypertension, several studies suggest that abnormal Cl^−^ transport is in fact the triggering mechanism.

Moreover, ABCC1 has been related to hypertension, modulating endothelial cell oxidative stress and it is known that this protein plays a critical role in the hypertensive response to angiotensin II [10], [51]. Although these two studies used ABCC1 knockout mice, the authors only studied the effect of this protein in endothelial cells. Nothing is known about the renal function in those mice. Our results showing that response of MA104 cells to Cl^−^ is not as regulated by urea as the cellular response to Na^+^ (Fig. 4) are in accordance with the results obtained by several studies, and the differences in ABCC1 regulation by NaCl we observed in cells with high and normal SSC point to a role of this protein in NaCl and urea handling by collecting duct cells. Therefore, the present study claims attention for the possible important role of ABCC1 in the distal nephron, which has been considerably disregarded.

The change in cell granularity obligated us to separate the cells in two regions, one containing cells with normal SSC and the other containing cells with high SSC. Doing this, we observed that NaCl reduced the percentage of cells expressing ABCC1 (Fig. 8) and reduced the efflux of the fluorescent dye in normal SSC cells. On the other hand, NaCl increased the percentage of cells expressing ABCC1 (Fig. 8) and the efflux of the dye (Fig. 10) in high SSC cells. Moreover, although urea did not reversed NaCl effects in normal SSC cells, it indeed reversed these effects in high SSC cells (Fig. 11).

It has been shown that ABCC1 exports both reduced and oxidized glutathione [34], [35] and may increase oxidative damage [10], [51], [52]. Moreover, GSH is known to stimulate the transport of several substances, such as estrone sulfate [53]. Our results, taken together, suggest that urea prevents the alterations leading to the increase of the high SSC subpopulation (Fig. 7, 8, 10 and 11), which seems to be the subpopulation having reduced cell growth, an effect also reversed by urea (Fig. 4).

Although the precise role of ABCC1 in this effect is yet not known, a possible explanation is a role in cell volume control in hyperosmotic medium. It is known that hyperosmolality leads initially to loss of intracellular volume and this can trigger the synthesis and transport of osmotic active solutes, such as sodium, taurin and betain [12], [54], [55]. Therefore, it is possible that ABCC1 has an important role in cell volume regulation, which remains to be defined. In accordance with this hypothesis, it is known that animals treated with DEM (which forms complexes with GSH, reducing its intracellular levels) showed severe urinary concentration deficiencies [12], [13] and that etoposide induced polyuria in abcc1(−/−) mice [14]. Lash and Tokarz [56] showed that 4-(2-thienyl) butyric acid produced GSH oxidation, lipid peroxidation, and inhibition of cellular respiration in cells from both proximal and distal rat renal tubules, which are indicative of oxidative stress and mitochondrial dysfunction. Guillermina et al [57] showed that mercuric chloride nephrotoxicity was markedly in rats depleted of GSH, and observed that the fractional excretion of water was increased in these rats. Recently, Quezada et al [58] showed that increased activity of ABCC1 in diabetic glomeruli is correlated with an inadequate adaptive response to oxidative stress. Since changes in cellular glutathione redox homeostasis is related to the initiation and/or propagation of cellular apoptotic cascade [59], the results reported here suggest that ABCC1 plays an important role in the distal nephron, probably by regulating the intracellular levels of glutathione and/or active osmolytes, allowing the epithelia of both medullar collecting duct and straight distal tubule to survive in the harmful conditions of a hypertonic medullary interstitium and controlling diuresis. Hence, the nephrotoxicity of several anticancer agents, such as cis-platinum, ifsofamide, methotrexate, citumixab and panitumumab, mitocin C and gemcitabine should be re-evaluated taken into account the possible role of ABCC1 in this process. For example, methotrexate is an antifolate drug and it is known that ABCC1 transports folate [60]. ABCC1 is also capable of transporting the glucoronide conjugate of etoposide and a GSH conjugate of doxorubicin [61], [62]. Our results suggest that any alteration in GSH status in collecting duct renal cells could negatively affect the ability of these cells to deal with their mechanisms of concentrating urine and thus more attention is needed to this part of the nephron when studying nephrotoxicity of new (and old) anticancer drugs.

### Ethical Standards

All experiments present in this manuscript were performed according to local legislation.
